# Optimized SNR-based ECAP threshold determination is comparable to the judgement of human evaluators

**DOI:** 10.1371/journal.pone.0259347

**Published:** 2021-11-01

**Authors:** Lutz Gärtner, Philipp Spitzer, Kathrin Lauss, Marko Takanen, Thomas Lenarz, Sebastian Hoth

**Affiliations:** 1 Department of Otolaryngology, Hannover Medical School, Hannover, Germany; 2 Research and Development, MED-EL Medical Electronics, Innsbruck, Austria; 3 Funktionsbereich Audiologie, Universitäts-HNO-Klinik, Heidelberg, Germany; Universidad de Chile, CHILE

## Abstract

In cochlear implant (CI) users, measurements of electrically evoked compound action potentials (ECAPs) prove the functionality of the neuron-electrode interface. Objective measures, e.g., the ECAP threshold, may serve as a basis for the clinical adjustment of the device for the optimal benefit of the CI user. As for many neural responses, the threshold determination often is based on the subjective assessment of the clinical specialist, whose decision-making process could be aided by autonomous computational algorithms. To that end, we extended the signal-to-noise ratio (SNR) approach for ECAP threshold determination to be applicable for FineGrain (FG) ECAP responses. The new approach takes advantage of two features: the FG stimulation paradigm with its enhanced resolution of recordings, and SNR-based ECAP threshold determination, which allows defining thresholds independently of morphology and with comparably low computational power. Pearson’s correlation coefficient *r* between the ECAP threshold determined by five experienced evaluators and the threshold determined with the FG-SNR algorithm was in the range of *r* = 0.78–0.93. Between evaluators, *r* was in a comparable range of 0.84–0.93. A subset of the parameters of the algorithm was varied to identify the parameters with the highest potential to improve the FG-SNR formalism in the future. The two steps with the strongest influence on the agreement between the threshold estimate of the evaluators and the algorithm were the removal of undesired frequency components (denoising of the response traces) and the exact determination of the two time windows (signal and noise and noise only).”The parameters were linked to the properties of an ECAP response, indicating how to adjust the algorithm for the automatic detection of other neurophysiological responses.

## Introduction

A cochlear implant (CI) is an auditory prosthesis used to restore hearing in people with severe to profound hearing loss. Its multi-electrode array is normally inserted into the scala tympani and makes use of electrical stimulation to excite the surrounding neuronal population. In turn, this neuronal population generates action potentials that propagate along the auditory nerve and usually leads to auditory perception and speech comprehension. A CI can also be used to record the electrically evoked compound action potential (ECAP), which represents the neural response from multiple auditory nerve fibers to an electrical stimulus. A typical ECAP response waveform consists of a negative (N) peak at a latency of (0.33 ± 0.04) ms after stimulus onset and a positive (P) peak at (0.66 ± 0.08) ms [1]. The morphology of an ECAP response may change in that peaks may become less pronounced or disappear. A double P peak was observed with a prevalence of 7–18% [2, 3]. The ECAP amplitude is defined as the difference in voltage between the N and P peaks of an ECAP response. The ECAP threshold describes the minimum electrical charge (which is the product of the phase duration and electric current amplitude) needed to evoke a detectable ECAP response. Above ECAP threshold, the ECAP amplitude increases with stimulus strength, thereby describing the ECAP amplitude growth function (AGF). The AGF usually follows a sigmoidal shape, saturating at high stimulus levels. Above an individual behavioral threshold level, which is not necessarily equal to the ECAP threshold, loudness perception will usually increase with stimulus level. For safety reasons, stimulus levels during ECAP measurements are limited to avoid unpleasantly loud sensations. Therefore, recordings of ECAP AGFs up to saturation are rare.

ECAP measurements are important for clinical diagnostic and long-term care of CI users. Clinical specialists use the ECAP threshold frequently [4], e.g., for long-term monitoring of auditory health, to verify implant functionality, or as guidance for programming the audio processor. The clinical routine sets additional requirements for determining the ECAP threshold because the approach must be accurate, safe, and fast in order to be useable intraoperatively as well as postoperatively in adults and children. Different paradigms for recording ECAP responses have been presented in the scientific literature and by CI manufacturers (for a review see, e.g., [5]). The methods of determining ECAP thresholds are either fully or semi-automated, or they are entirely based on manual identification of ECAP responses by an experienced clinician. An automated system based on a decision-tree approach [6], named AutoNRTTM (Cochlear Ltd.), aims at simulating the procedure of visual detection by an expert. It has been implemented into the clinical software Nucleus Custom SoundTM Suite since 2005. A reliable N- and P-peak detection is a prerequisite for this method. Another method of threshold estimation is based on the linear extrapolation from the steepest portion of the AGF to the abscissa (stimulus level) with the intersection point representing the ECAP threshold. In fact, this approach has been exploited in clinical software for automated threshold determination by all CI manufacturers. Since each single ECAP response is contaminated with noise, averaging several responses at a given stimulus level is commonly used for denoising. In order to keep the measurement duration within reasonable time limits, only responses at discrete levels with a relatively big current step size were recorded. This limits the resolution at which the ECAP threshold can be determined. This problem was addressed in the design of the FineGrain stimulation paradigm [7], which samples the AGF in smaller stimulus steps and thus provides the means to use the higher ECAP AGF resolution for more accurate ECAP threshold determination. As a further development of this idea, FineGrain was combined with an automatic threshold determination approach, available as AutoART in the MAESTRO software (MED-EL Medical Electronics, Innsbruck, Austria) since 2017, version 7 and higher [7, 8].

ECAP measurements are important also for fundamental research on CIs. Due to regulatory and design requirements, the clinical software of any given manufacturer can only communicate with the CIs of that company. Therefore, the above-mentioned automated algorithms (AutoNRT^™^ and AutoART), imbedded in the clinical software, may not be ideally suited for research groups whose potential study populations cover several CI brands. In order to perform identical measurements for all study participants, regardless of the CI brand, the researchers may use (custom-made) research software that can communicate with all CIs via corresponding programmable research interfaces [9]. However, the advanced ECAP measurement paradigms of the clinical software cannot be used in the research setting. Especially for such investigations, analysis of the signal-to-noise ratio (SNR) provides an intriguing alternative to estimate the ECAP threshold in a fully automated manner and independently of the ECAP AGF. The SNR relates a desired signal (here: ECAP response) to background noise–a high SNR (above an application-specific level) means there is a high level of signal and a low level of background noise, which can be exploited for signal detection. Specifically, SNR-based ECAP threshold estimation can be achieved in one of two ways: (1) estimation can be based on the post-average residual noise and the useful variance [10] or (2) by a simple comparison of variances calculated for two different time windows within one recording. Hereby, the variance within that part of the recording window that potentially contains an ECAP response is compared to the variance within another part of the recording window that is known not to contain an ECAP response [11]. There are two advantages of the latter method. Firstly, a reliable threshold can be determined independently of the morphology of the ECAP response. As mentioned above, responses with double positive peaks may occur in up to 18% of cases [2, 3]. ECAP responses and algorithms based on peak-picking might struggle to define a reliable AGF if double peaks are registered. Secondly, the SNR algorithm itself requires only a comparably low computing power, which may be an advantage for certain applications.

The aim of this study was to make the benefits of advancements in clinical paradigms accessible to SNR-based estimations of the ECAP threshold. Traditional stimulation with discrete levels and large step-size was used for the SNR method [11]. However, it is likely that the precision of the approach would equally benefit from a higher resolution of the ECAP responses, as previously seen in AGF-based approaches. The novel FineGrain-SNR (FG-SNR) formalism is an extension of the SNR approach [11], which allows for single ECAP responses like those used with the FineGrain stimulation paradigm [7]. Here, we show that ECAP thresholds calculated with the FG-SNR algorithm are comparable with the thresholds determined by experienced clinicians. This, along with the fact that the FG-SNR algorithm is independent of any hardware requirements, also highlights the applicability of the algorithm to scientific investigations. In addition, we investigated which of the parameters of the FG-SNR algorithm are most important for future optimization. The findings of the investigation can be used for adjusting the FG-SNR algorithm for automatic threshold detection of other (electro-)neurophysiological responses.

## Methods

In order to reach the aims of the study, ECAP AGF measurements were first performed on voluntary CI users during their regular clinical appointments. Subsequently, the ECAP AGFs were analyzed by human evaluators to obtain data against which the decisions of the FG-SNR algorithm were compared and analyzed.

### Ethics statement

The Ethics committee of the Hannover Medical School, Germany, where the data were collected with the research software, approved the study (ID 6586). All participants provided their written informed consent before the start of any study-specific procedures. All participants have also given written informed consent to publish the respective data.

### Participants

Ten adult CI users (P01–P10) with a total of thirteen implants (participants P02, P03, and P05 were bilaterally implanted and provided data from both sides), contributed to this study. Demographic data are shown in Table 1. All measurements were conducted between October and December 2016. Only adults were included in this study. Another inclusion criterion was that a CI with an i100 platform manufactured by MED-EL Medical Electronics (Innsbruck, Austria) had to be in use for compatibility with the ECAP measurement hardware and software. Users were asked during their regular follow-up visit if they would agree to participate in the study. There was no additional criterion for selecting a participant. No additional visit was necessary for the measurements.

**Table 1:** Participant demographics.

Implant ID	Age at implantation in years	Age at measurement in years	Side of implantation	Gender	Implant type	Electrode array type
**P01**	55–60	55–60	R	F	SYNCHRONY	FLEX24
**P02L**	60–65	60–65	L	M	SYNCHRONY	FLEX28
**P02R**	60–65	60–65	R	M	SYNCHRONY	FLEX28
**P03L**	50–55	50–55	L	F	SYNCHRONY	FLEX24
**P03R**	50–55	50–55	R	F	SYNCHRONY	FLEX24
**P04**	55–60	55–60	R	M	SYNCHRONY	FLEX28
**P05L**	45–50	50–55	L	F	SYNCHRONY	FLEX24
**P05R**	50–55	50–55	R	F	SYNCHRONY	FLEX24
**P06**	65–70	65–70	L	F	SYNCHRONY	FLEX28
**P07**	50–55	50–55	L	M	SYNCHRONY	FLEX28
**P08**	55–60	55–60	L	F	SYNCHRONY	FLEX28
**P09**	55–60	55–60	L	F	SYNCHRONY	FLEX24
**P10**	45–50	50–55	R	F	CONCERTO	STANDARD

Age ranges are used instead of explicit ages to avoid potentially identifying participant information.

### ECAP measurements

Measurements were recorded via the MAX Programming Interface (MED-EL Medical Electronics, Innsbruck, Austria) connected to a personal computer running the research software that is described in [7]. Symmetric charge-balanced biphasic pulses were used to elicit the nerve response. The two phases were separated by an interphase gap of 2.1 μs. Stimulus rate was between 40 and 76 pulses per second (pps). Pulse duration was between 30 and 40 μs. The delay time between stimulus onset and the beginning of the recording was between 125 and 145 μs. Responses from anodic and cathodic leading stimuli were averaged to reduce the stimulus artifact according to the alternating polarity paradigm (e.g., [5]). The results from each implant comprised amplitude growth data recorded with the FG paradigm for all 12 electrode contacts. This corresponds to 156 AGFs available for analysis.

### Evaluation of the ECAP measurements by clinicians

Five experienced clinicians (AD, KS, LG, PS, SS) and one inexperienced clinician (SK), all hereafter referred to as “evaluators”, analyzed each of the 156 AGFs independently from each other in a randomized order (i.e., the order was different for each analyst). All traces of one stimulation/recording electrode pair were available in the order of stimulus intensity. In addition, the custom software, ART Analyzer, was made available to the evaluators. This graphically orientated tool allows clinicians to apply various artifact reduction methods (alternating stimulation, zero amplitude template subtraction, scaled template subtraction) and includes an extrapolation tool that can optionally be used for threshold determination. The evaluators judged whether an ECAP signal was present and, if yes, specified the ECAP threshold. The evaluators were free to choose their own methods and criteria for classification and threshold determination. Signals classified as “ECAP response unsure” by an evaluator were considered as “no ECAP response”.

### FG-SNR algorithm

The FineGrain (FG) stimulation paradigm usually does not repeat stimulation at the same level. Consequently, its single ECAP recordings contain more noise, which could compromise the success of the SNR approach, as already described in [11]. For that reason, the extension of the SNR approach in order to cope with the FG stimulation paradigm requires the definition of a data processing pipeline (DPP) for the ECAP tracings. The main task of the DPP is to maximize the accuracy of ECAP threshold determination by reducing artifacts and noise. This section gives an overview of the functionality of the FG-SNR algorithm, which is explained in greater detail in S1 Appendix.

**Fig 1:**
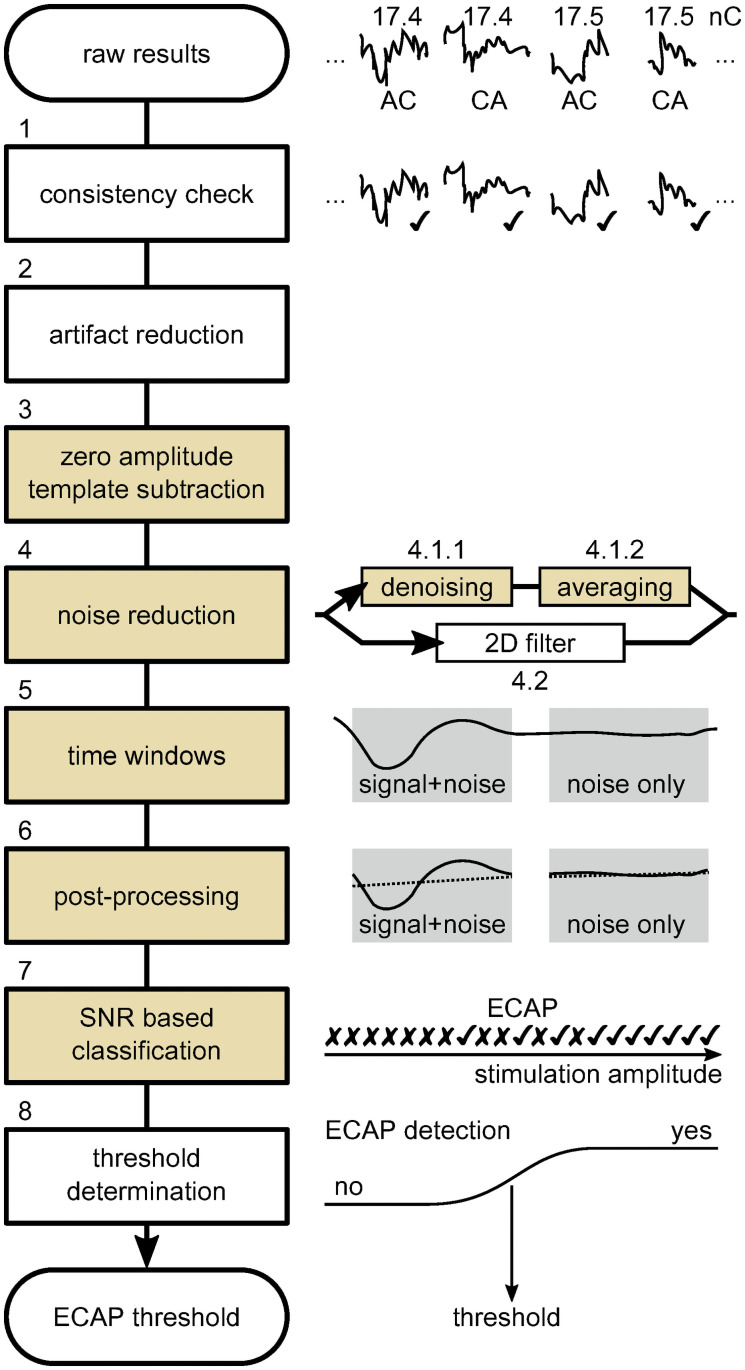
Data processing pipeline of the FG-SNR algorithm. AC: anodic-cathodic; CA: cathodic-anodic. The colored boxes represent steps where different parameters are being evaluated.

The workflow of the FG-SNR algorithm is illustrated in Fig 1. First, the responses are checked for consistency (i.e., clipped responses are excluded), after which the alternating-polarity paradigm is used to minimize stimulation artifacts. Then, a “zero amplitude template” (ZAT), evoked by a stimulus of vanishing amplitude, is subtracted from the responses before a two-stepped noise reduction is applied to attenuate undesired frequency components (artifacts and noise) and to reduce noisiness of single ECAP traces. Once the ECAP recordings have been denoised, ECAP threshold determination works as described in [11]. A quantity *var* (variance) was introduced, which is the mean square amplitude of a signal, *y*, within a time window [*t*_i_; *t*_j_]. Two time windows were under consideration. One time window represents the response with residual noise, “signal+noise”, within the time window [*t*_1_; *t*_2_] and the other represents the “noise only” part within [*t*_3_; *t*_4_]. Thus,

$${var}_{signal+noise}=\frac{1}{t_{2}-t_{1}}\int_{t_{1}}^{t_{2}}{\left(y-y_{ref}\right)}^{2}dt$$

and

$${var}_{noise}=\frac{1}{t_{4}-t_{3}}\int_{t_{3}}^{t_{4}}{\left(y-y_{ref}\right)}^{2}dt.$$

**Fig 2:**
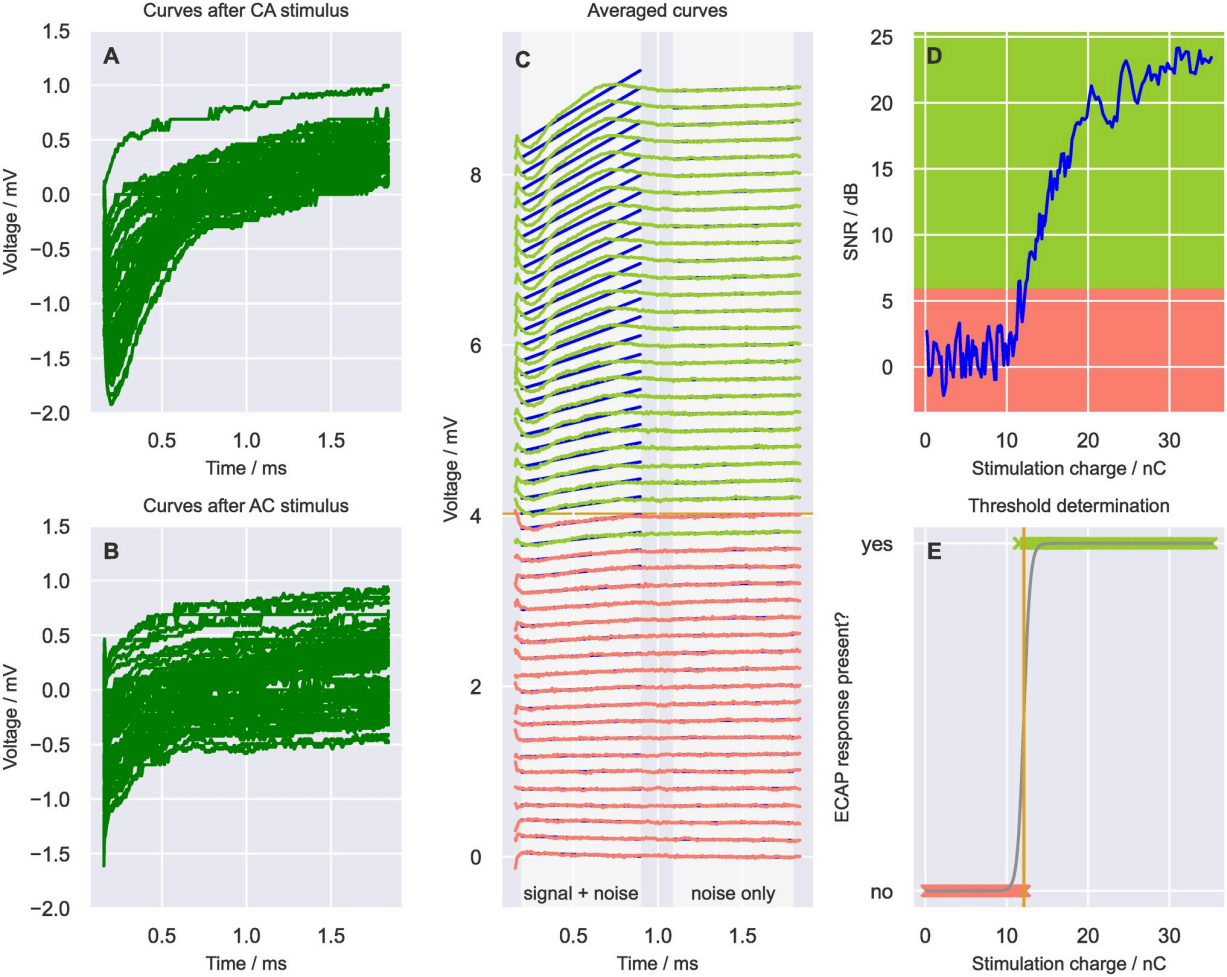
Example result of an ECAP measurement obtained from the FG-SNR algorithm within a stimulation range of 0 to 36 nC. For illustrative purposes, only every fourth curve is shown in panels A (cathodic-anodic), B (anodic-cathodic), and C (curves after averaging and ZAT subtraction). In panel C, green traces indicate responses with an ECAP signal and red traces those without. Straight blue lines (partly hidden behind the actual data) indicate the first order polynominal fit separately applied to the “signal + noise” and “noise only” time windows. Data from participant P03L, stimulating electrode E01, recording electrode E03.

The reference line, *y*_*ref*_, is a function fitted to the data to compensate for the stimulus artifact and possible DC components. Subsequently, the quotient

$$q=SNR=\frac{{var}_{signal+noise}}{{var}_{noise}}$$ (1)

is derived and finally the stimulus level dependence (i.e., the AGF) of *q* is analyzed. The response threshold is defined by the crossing of this function with the horizontal line, *q*_0_ (see Fig 2D, where *q*_0_ = 6 dB). If the solution is ambiguous, i.e., more than one crossing, the values of *q* are converted to a binary variable (0 or 1) that indicates the absence (0 if *q* ≤ *q*_0_) or presence (1 if *q* > *q*_0_) of a response. Next, a sigmoid discrimination function of Boltzmann type

$$f\left(x\right)=\frac{1}{1+e^{-k(x-x_{0})}}$$

was fitted (see Fig 2E) using the Levenberg-Marquardt algorithm [12]. Here, *x* denotes stimulus strength, *f* is the binary AGF, and *x*_0_ (inflection point of the sigmoid) and *k* (parameter which corresponds to the slope of the sigmoid at the inflection point) are the parameters to be fitted. In the FG-SNR algorithm, *x*_0_ is used to denote the ECAP threshold.

Fig 2 summarizes the different steps in the DPP which were carried out with baseline parameter values (see Table 2) in an individual case. Quasi-continuous FG stimuli were applied with alternating polarity, which resulted in unprocessed (raw) cathodic-anodic (A) and anodic-cathodic (B) recording curves. Five recordings obtained from adjacent stimuli were averaged. Recordings below or equal to a stimulus charge of 5 nC were averaged to estimate a ZAT and subtracted from each averaged resultant curve shown in C. Next, “signal + noise” and “noise only” time windows of each averaged curve were defined. If the SNR was above threshold, it was classified as “ECAP signal present” (green traces, C). The SNRs, according to Eq (1), were plotted as a function of the stimulus charge (“SNR growth function”; D) and a sigmoidal fit was applied to determine the ECAP threshold (E).

**Table 2:** Adjustable parameters of the DPP.

Parameter	Brief description	Baseline value
**Zero Amplitude Template (ZAT) option**	Compensate for switch-on artifact of the amplifier. Only artifact is assumed up to a maximum charge of:	5 nC
**Low-pass filter edge frequency**	Eliminate high-frequency interferences of single ECAP responses	3 kHz
**Number of curves averaged**	Reduce noise by averaging adjacent ECAP responses of slightly different stimulus levels	5
**Time window of the “signal + noise” part**	Define the time window where ECAP response is expected	195–895 μs
**Time window of the “noise only” part**	Define the time window where no ECAP response is expected	1095–1795 μs
**Method of post processing**	Compensate for stimulus artifact	1^st^ order polynomial fit
**SNR ratio q** (**see** Eq 1)	To distinguish “signal+noise” from “noise only”	6 dB

### Investigation of optimal parameters

All raw data were processed with the novel FG-SNR algorithm using the values of the set of baseline parameters (Table 2) to determine whether an ECAP response was present. In case an ECAP response was present, the ECAP threshold was estimated. Outcomes of threshold determination deduced from the algorithm were compared to those of the evaluators. As listed in Table 2, the DPP contains several parameters that can be adjusted to optimize its performance for future applications. Here, we varied the values of each parameter independently to explore their influence and to find the optimum with respect to the correlation with the evaluator’s decisions. A detailed description of how the parameters were varied is given in S1 Appendix.

### Statistical analysis

The performance of the algorithm was assessed by means of both descriptive statistics and statistical tests. The impacts of the parameter variations were also assessed using descriptive statistics. Pearson’s correlation coefficient *r* was used as the primary descriptive statistical parameter to capture similarity between the threshold estimates determined by the algorithm and the evaluators. However, this outcome is affected by the number of samples. For that reason, the number of AGFs, which were classified as containing an ECAP by both the FG-SNR algorithm and any individual evaluator, was used as secondary descriptive parameter. The statistical tests consisted of the analysis of variance (ANOVA) procedure for (generalized) linear mixed-effects models to assess the performance of the FG-SNR algorithm. The validity of the assumptions underlying such models [13–15], i.e., normality of the residuals and the normality of the random effects, was always verified both by means of statistical testing [16] with the significance level of 5% and by means of a visual comparison of the distributions and the quantiles against their theoretical counterparts. Upon discovery of significant effects, planned pairwise comparison of means were performed to gain insight into the nature of the effect using false-discovery rate-based compensation for multiple comparisons [17].

Several statistical tests were performed to investigate the sensitivity and bias of the FG-SNR algorithm in detecting the presence of an ECAP response. To that end, an ANOVA procedure was first performed on a binomial generalized linear mixed-effects model. The dependent variable in this model was the algorithm’s decision (1: ECAP present, 0: no ECAP present). The evaluator’s decision (1: ECAP present, 0: no ECAP present) and ID (AD, KS, LG, PS, SK and SS) were included as fixed factors, and the implant ID (P01, P02L, P02R, P03L, P03R, P04, P05L, P05R, P06, P07, P08, P09 and P10) was included as a random factor. An additional signal-detection theory-based analysis was then performed to address the sensitivity and a potential bias of the algorithm’s decision using data pooled across the evaluators. To that end, AUC values (AUC is the area under the curve derived from the receiver-operator characteristics) and criteria were computed from the hit rate (both the algorithm and the majority of evaluators indicated an ECAP threshold was present) and false alarm rate (the algorithm indicated an ECAP response but the evaluators did not). Subsequently, separate ANOVA procedures were performed on two linear mixed-effects models that both contained the implant ID as a random factor and had either the AUC value or the criterion as the dependent variable.

The other set of statistical tests investigated if and how the ECAP threshold estimates provided by the algorithm differ from those provided by the evaluators. To that end, the differences between the ECAP threshold estimates of the algorithm and of any individual evaluator were extracted. Upon extracting the differences, a linear mixed-effects model was constructed by having the difference as the dependent variable and by including the evaluator ID and stimulating electrode (from 1 to 12) as fixed factors and the implant ID as a random factor. Finally, an ANOVA was performed to investigate the effects of the fixed factors on the aforementioned difference.

## Results

**Fig 3:**
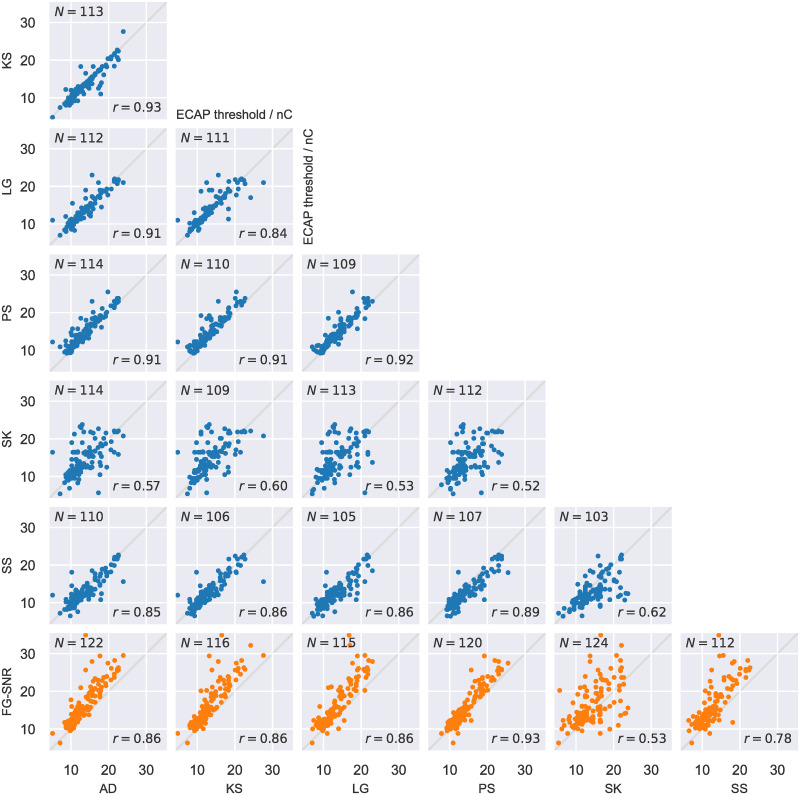
Comparison of ECAP threshold determination by the evaluators and the FG-SNR algorithm. Results of the FG-SNR approach were calculated with baseline parameters. Underlying data is shown in S1 and S2 Tables.

The outcome of the FG-SNR algorithm with baseline settings was compared to thresholds determined by six evaluators. More specifically, we looked at (dis)similarities in classification of ECAP presence and between thresholds. The FG-SNR algorithm and all six evaluators agreed in their classification of ECAP presence or ECAP absence in 64% (100/156) of the recordings. In 93 cases (59.6%), all evaluators and the algorithm agreed on the presence of an ECAP response, and in seven cases (4.5%) all agreed on its absence. In the remaining 56 cases (36%), results differed amongst the evaluators or between evaluators and the algorithm. With respect to ECAP threshold accuracy, the results obtained with the algorithm correlated well with the five most experienced evaluators’ assessments. Pearson’s correlation coefficient *r* was between 0.78 and 0.93 (Fig 3). ECAP thresholds determined by the least experienced evaluator, SK, were less consistent with the algorithm (*r* = 0.53). However, comparing SK’s decisions with those of the five experienced evaluators resulted in an equally moderate correlation (*r* = 0.52–0.62).

**Fig 4:**
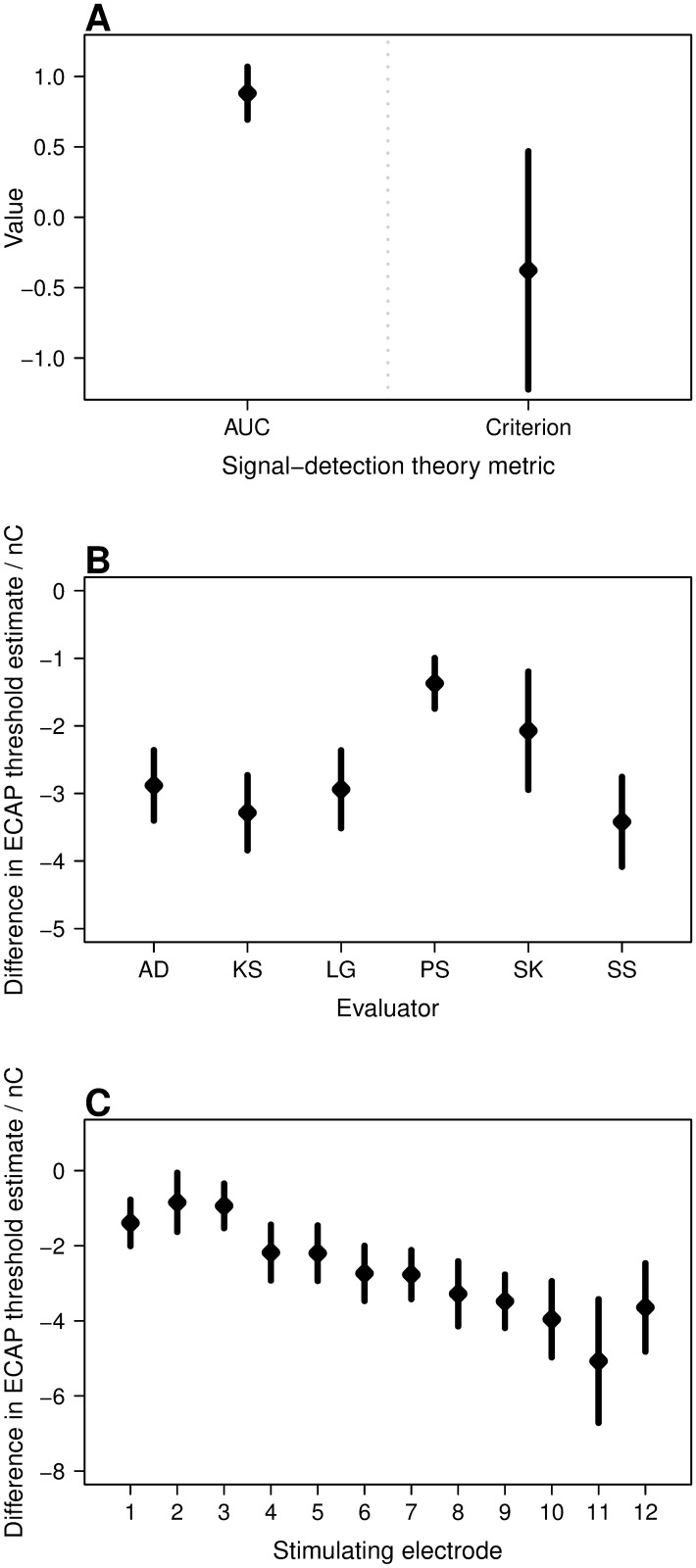
Marginal means and their 95% confidence intervals. A. For outcome values from signal-detection theory-based analysis. B and C. For differences in the ECAP threshold estimates of the FG-SNR algorithm and of the human evaluators. Panel B shows the difference to individual human evaluators, averaged across all implants and stimulating electrodes. Panel C shows the differences for different stimulating electrodes averaged across all implants and evaluators. Data underlying these panels (A-C) are given in S3 Table.

The first statistical test that was performed revealed that the algorithm’s decision (ECAP presence: yes/no) depended, with high significance, on the evaluator’s decision (*χ*^2^ ≈ 42.0, *df* = 1, *p* < 1 e-10). This result was in accordance with the high correlation found between the ECAP thresholds determined by the algorithm and by the evaluators. The subsequent signal-detection theory-based analysis bolstered this finding by revealing only a highly significant interceptor effect (*F*[1, 13] ≈ 243.8, *p* < 0.001) for the AUC, but no significant effects for the criterion (*F*[1, 13] ≈ 2.2, *p* > 0.05). Fig 4A illustrates this by showing the marginal means and their 95% confidence intervals for the outcomes of the signal-detection theory-based analysis. Firstly, the AUC of the algorithm is on average around 0.88, which corresponds to an excellent discrimination ability [18]. In other words, the ability of the algorithm to discriminate between the presence and absence of an ECAP amongst the traces is similar to the majority decision reached amongst the evaluators. Secondly, the average criterion of -0.38 indicates that the algorithm has a small, albeit statistically non-significant, tendency to classify an ECAP as present when the majority of the evaluators speak against it.

The difference in ECAP threshold estimates between the algorithm and individual evaluator was significantly affected by the evaluator ID (*χ*^2^ ≈ 123.6, *df* = 5, *p* < 1 e-10) and the stimulating electrode (*χ*^2^ ≈ 35.0, *df* = 11, *p* < 0.001). The graphs in Fig 4B and 4C illustrate these effects, where the marginal means and their 95% confidence intervals show that the ECAP threshold estimate of the algorithm was on average approximately 2.7 nC higher than those estimated by the evaluators (Fig 4B). Moreover, the threshold estimates differed between the evaluators (Fig 4B), which reflects the diversity in the evaluators’ opinions about the definition of an ECAP threshold. Here, the threshold estimates of evaluator PS were found to be the closest to the estimates of the algorithm, while the ones of evaluator SS were found to differ the most from the estimates provided by the algorithm (*p* < 0.001 for paired comparison between evaluators PS and SS). The effect of the stimulating electrode is shown in Fig 4C, which demonstrates that the algorithm’s ECAP threshold estimate was closest to the estimates of the evaluators for the most apical electrodes (electrodes 1, 2, and 3) and differed the most for the basal electrodes (*p* < 0.05 for all paired comparisons between electrode 11 and electrodes 1, 2, and 3). The dependence of the difference in ECAP threshold estimates (between evaluators and algorithm) on the stimulating electrode can be explained by the SNR in general being higher for apical electrodes [3, 19], which facilitates the determination of ECAP thresholds for both the algorithm and the evaluator.

### Impact of parameter variations in the DPP

**Fig 5:**
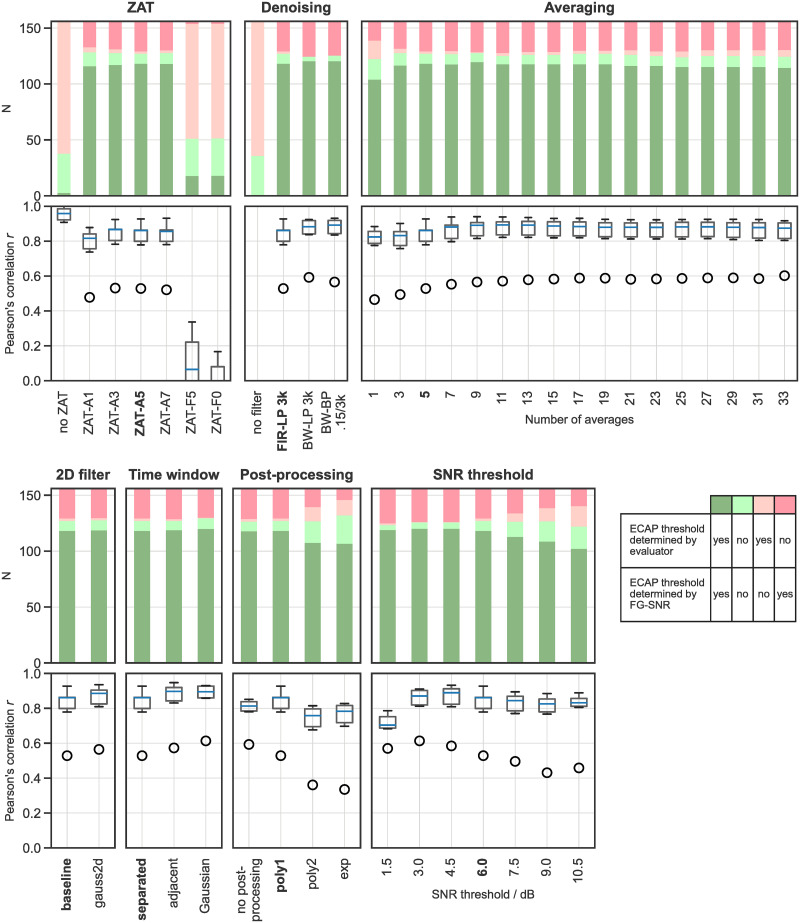
Effect of choosing different parameter options on ECAP response classification and threshold determination. The different parameters are shown on top of each block: ZAT, denoising, averaging, 2D filter, time window, post-processing, SNR threshold.

The results from evaluating different parameters are shown with similar graphs in Fig 5. For each parameter, the horizontal axis shows the different options for varying its value. The label for the baseline condition appears in bold font. The stacked bars in the topmost panel for each parameter indicate the number of AGFs where the algorithm and the “average” evaluator (the mean value of the 6 evaluators’ opinions) agreed or disagreed in the ECAP classification. The cases where the evaluators and the algorithm agreed on ECAP presence (dark green) were used to compute Pearson’s *r* for the ECAP thresholds, shown as a boxplot presentation of medians and quartiles in the bottommost panel for each parameter. Circles are indicating outliers. Here, all outliers stem from the poorer agreement between the unexperienced evaluator SK and the FG-SNR algorithm. All results are also presented in S1 and S2 Tables. A summary of the tested parameter options and the optimal parameters is given in Table 3.

**Table 3:** Summary of all varied parameters, their tested conditions, and the “best” option.

Parameter	Baseline value	Tested Conditions	Best option
**Zero Amplitude Template (ZAT) option**	5 nC	No ZAT; averaging below 1, 3, 7 nC; F5 and F0 fit	5 nC
**Denoising options**	3 kHz (FIR-LP 3k)	No filter, Butterworth filter lowpass 3k and Butterworth Bandpass 0.15/3k	Both Butterworth filters
**Averaging options: Number of curves averaged**	5 curves	1,3,7,9,11,13,17,19,21,23,25,27,29,31, 33	9
**Time window options: “signal + noise” and “noise only” part**	Separated	Adjacent	Adjacent and Gaussian
		Gaussian	
**Post processing options**	First order polynomial fit (poly1)	No post-processing, second order polynomial fit (poly2), exponential fit (exp)	Poly1
**SNR threshold criteria q** (**see** Eq 1)	6 dB	1.5, 3, 4.5, 7.5, 9, 10.5 dB	6 dB

Cases where (one or more) conditions outperformed the baseline setting are highlighted in green. For details on the tested conditions, refer the corresponding section in the results or the S1 Appendix.

#### Application of different zero amplitude template (ZAT) options

Omitting the subtraction of a ZAT can be seen to result in a much lower fraction of agreement on ECAP presence. When no subtraction was applied, the algorithm missed the majority of cases that were classified as “ECAP response present” by the evaluators. The fraction of cases where the evaluators and the algorithm agreed was very small, with high values of r. However, this is not meaningful for such a low number of data points.

All ZAT-A options are based on averaged sub-threshold recordings, where A stands for averaged below the given stimulus level in nC. While the distribution of classified fractions is similar across all ZAT-A options, ZAT-A1 slightly reduces *r* compared to other options where higher numbers of curves are averaged (ZAT-A3, ZAT-A5, ZAT-A7).

Approaches to represent the ZAT as fit (ZAT-F5, ZAT-F0) lead to a lower fraction of detected ECAP thresholds and lower values of *r* compared to any approach comprising of “averaging only”. The polynomial function of 2nd order (ZAT-F5) was not able to represent the ZAT well enough. In the initial part of the curve (within the time window “signal + noise”), an artifact component was not reduced and, therefore, most traces were classified as ECAPs regardless of whether they actually contained one or not. That made it impossible for the algorithm to determine an ECAP threshold. In summary, none of the options that subtract the ZAT based on the chosen fit nor the options that tested averaging over different stimulus intensity ranges were considered an improvement compared to the baseline condition (ZAT-A5 with averaging curves between 0 and 5 nC).

#### Application of different denoising options

No thresholds at all were determined by the algorithm when filtering was omitted. Pearson’s r was similarly high for all three filters that were tested, however, the lowpass (LP; cutoff at 3 kHz) and bandpass (BP; passband between 0.15 and 3 kHz) Butterworth (BW) infinite impulse response (IIR) filters (BW-LP 3k and BW-BP 0.15/3k, respectively) resulted in a slightly higher r for two evaluators. Thus, both Butterworth filter options can be considered an improvement over the LP finite impulse response (FIR-LP 3k) filter used as the baseline option.

#### Application of different averaging options

The highest r of ECAP threshold estimation between the algorithm and the evaluators was found when seven or more curves were averaged. No averaging at all led to the lowest r with most evaluators. Correlation remained stable even when a high number of curves were averaged, although averaging ECAP responses at different stimulus levels is expected to decrease the accuracy of threshold estimation. Therefore, we aimed to identify the averaging option with the lowest number of adjacent response curves that gives the best results: nine curves. This option was considered an improvement over the baseline parameter of averaging five responses.

#### Use a 2D filter instead of separate denoising and averaging

A 2D Gaussian filter kernel with a sigma of 3 and 5 samples in the respective directions was used. Although the parameter choice was not systematically optimized, both the resulting r and the fraction of agreement in classification were similar to the baseline conditions with a tendency to be better in most cases. Our implementation required fewer lines of code and was executed slightly faster (numeric results are not presented here because the implementation was not optimized for speed).

#### Application of different time windows

Pearson’s r was highest for defining “signal + noise” and “noise only” parts with the *adjacent* and the *Gaussian* time windows. Both options were considered an improvement over the baseline setting, *separated*. In other words, the performance of the algorithm was improved when the time windows for the “signal + noise” and “noise only” parts were either extended so that together they cover almost the whole ECAP recording window (adjacent), or extended even to overlap with each other and weighted with Gaussian functions (Gaussian).

#### Application of different post-processing options

The first order polynomial poly1 option (baseline setting) to remove residual stimulation artifact resulted in the highest value of r. The algorithm seems to be robust against omitting the post processing because the results are highly similar for the no processing option as well. However, using either a second order polynomial poly2 or an exponential exp fit in post processing decreased r.

#### Application of different SNR threshold criteria

A SNR threshold of 6 dB lead to the highest value of r. The advantages of the 6 dB cut-off are that it is widely used as a criterion in signal detection applications, it has been tested before [11], and the original arguments still hold: with 6 dB, the energy of a detected ECAP signal is four times higher than the energy of the background noise [11]. A less rigorous criterion, e.g., 3 or 4.5 dB, would result in signals with smaller SNR to be classified as ECAPs, which might result in more false positives (our data show this effect for the SNR threshold option of 1.5 dB).

## Discussion

The SNR threshold detection algorithm [11] has the advantage of operating at relatively low ECAP amplitudes and working for different ECAP response morphologies. The FG stimulation paradigm [7] provides the means to sample the ECAP AGF with high resolution. The novel FG-SNR approach allows for an extension of the SNR algorithm regarding the peculiarities of the FG paradigm and was evaluated in this study. It was not the aim of this study to compare or rank different methods of ECAP detection and threshold determination.

A new threshold detection algorithm must ultimately be evaluated versus the classification by a clinical specialist [20], because this represents the only standard for “true” ECAP thresholds to date. Since a certain bias–due to the individual methodology of the evaluator–is expected to influence their opinion, we consulted six evaluators in attempt to understand not only the performance of the FG-SNR approach, but also the variation in judgement across individual specialists. We hypothesized that this validates the FG-SNR approach for use in clinical practice. We found that the FG-SNR approach with baseline parameters reflects the choice of human evaluators with regard to ECAP presence. The best correlation between the ECAP determination of the evaluators was *r* = 0.93. Therefore, it is remarkable that the best correlation between the algorithm (using baseline parameters) and the evaluators was *r* = 0.93 as well. Thresholds determined by the evaluators and the algorithm were closest to each other at electrode contacts located at the apical end of the electrode array and differed the most from each other at contacts located on the basal side (Fig 4B and 4C). This is in agreement with ECAP amplitudes and slopes being largest at apical and lowest at basal electrode contacts in MED-EL CI users [3, 19, 21] and indicates that estimating the threshold is more challenging in the basal region for the evaluators, for the FG-SNR algorithm, and for both. This might be due to more recording noise in the base of the cochlea, possibly due to contacts being located further away from neural structures or a lower density/survival of neurons compared to the apex.

Any (automatic) ECAP threshold estimation must inevitably address the question of how to determine the threshold. Some clinicians prefer the method of “first visual” to denote the stimulus strength corresponding to the first trace they deem to contain a valid ECAP response as the ECAP threshold. Others use the method of interpolation and define the ECAP threshold as the stimulus strength at which ECAP AGF intersects with the noise floor. Among the automatic ECAP measurement algorithms, AutoNRT^™^ can be seen to mimic the approach of “first visual” whereas AutoART follows the method of interpolation. The present FG-SNR algorithm and the previously presented SNR algorithms [10, 11] are, in our opinion, closer to the method of “first visual”. Since the FG-SNR algorithm bases the ECAP threshold determination on a sigmoidal fit on binary (ECAP yes/no) classifications instead of fitting a function to the ECAP AGF, the FG-SNR algorithm could theoretically detect the ECAP threshold at a lower stimulus strength than clinicians or algorithms relying partially on the interpolation. However, this remains have to be evaluated by presenting only a subset of the ECAP AGF measurements to the FG-SNR algorithm and searching for the stimulus strength, to which the ECAP threshold estimates provided by the algorithm converge. In addition, it should be noted that the “first visual” approach and algorithms of determining the ECAP threshold cannot estimate the ECAP AGF slope.

Differences in experience amongst evaluators affect the degree of similarity between the evaluators’ and the algorithm’s decision. Clinicians continuously improve their craft in ECAP threshold determination and ECAP detection with experience. In [22], when evaluating AutoNRT^™^ algorithm (Cochlear Ltd.), the ECAP classification and threshold determination of human evaluators were used as a reference as well (five experienced and two less experienced evaluators in that case). The deviations observed amongst these seven evaluators are not directly comparable to our study since other stimulus scales were used. However, similarly to our study, the less-experienced evaluators tended to deviate more from the “average evaluators” estimate than the algorithm that was under review. One limitation of our current study may be that only one unexperienced evaluator was recruited to analyze the ECAP responses. Based on the experience published earlier [22], we did not expect that unexperienced evaluators would add significant value to this study. However, we wanted to show that detection of ECAP responses and estimating their thresholds needs training. The experienced evaluators had worked a few years up to 20 years in the field of cochlear implants whereas the unexperienced evaluator had been involved only a few months by the time the evaluators analyzed the ECAP responses.

We varied different parameters of the DPP of the FG-SNR approach separately to observe and understand the effects on the outcome measure. The procedures and parameters which we found to have the highest potential to alter or improve the DPP were (1) denoising of the response traces, (2) selection of the time windows for the “signal + noise” and “noise only” parts, and (3) the number of ECAP traces used for averaging. The first two most important parameters are closely related to the neurophysiology-based knowledge about the characteristics of the target response (the ECAP in this case). This is encouraging for applying the FG-SNR algorithm to the automatic detection of other neurophysiological responses. The importance of the averaging step highlights the need to adjust the number of traces depending on the noisiness of the single measurement and the applicable step size when using the FG stimulation paradigm to record a given neurophysiological response.

Denoising of the response traces is important for the functionality of the FG-SNR algorithm because its purpose is to preserve signal components in the frequency range of a potential ECAP response while removing and/or attentuating undesired components (i.e., artifacts and noise). The better the denoising filter fulfils this purpose, the easier the classification becomes. Indeed, from Fig 5 it becomes obvious that FG-SNR could not detect a single ECAP response when no filter was applied. However, if the filter is too sharply tuned to match the frequency properties of the ECAP response, the FG-SNR algorithm becomes overly sensitive and could classify any remaining signal as an ECAP. For practical applications, the IIR filters are perhaps more interesting since they offer lower computational costs to achieve a desired frequency response. The uncontrollable phase response of an IIR filter does not affect the performance of the FG-SNR algorithm, but the IIR filters had to be applied backwards to minimize the influence of ringing artifacts in our study. In its final portion, the ECAP signal was approximately flat and, therefore, initial conditions for the filter were easily determined.

Appropriate determination of the time windows for the “signal + noise” and “noise only” parts is vital for the functionality of the FG-SNR algorithm. The two time windows are to be selected so that the former contains the “meaningful variance” of the response and the latter contains only the measurement noise (compare to Eq 1). It was shown that the latencies of the N and P peaks of the ECAP are only mildly dependent on the stimulus strength. The cohort of N and P latencies were observed to be in the regions 300–400 μs and 600–700 μs, respectively, and independently of the stimulus charge and electrode [1]. However, the “signal + noise” window contains not only the response but, among others, also residual stimulation artifacts and ringing artifacts from the denoising step that can interfere with the SNR calculation. In the original SNR approach [11], the “signal + noise” and “noise only” parts are both extracted from different regions of the same curve. This has the benefit of saving measurements (and, therefore, most likely time) compared to the task of generating two separate recordings for noise and signal. Moreover, it ensures that the ambient noise and recording conditions are identical. Consistent with this theorem, we found that, e.g., temporal weighting of the components within the “signal + noise” and “noise only” parts with a Gaussian function can improve the performance (median *r* increased from 0.86 to 0.89), because the stimulation and ringing artifacts are more prominent at the beginning and at the end of the response.

Averaging of the adjacent ECAP traces to reduce noise was also found to impact the performance of the FG-SNR algorithm. This can be explained by the noisiness of a single ECAP measurement, which should be minimized by averaging across several recordings. When the FG stimulation paradigm [7] is used to perform the ECAP measurements, the stimulus charge is monotonously increased in small steps. Above threshold, the amplitude of the resulting response is also expected to increase. Averaging of a certain number *n* of consecutive responses should reduce noise while the error introduced by different ECAP amplitudes is limited when *n* is small. An uneven number of averages ensures that an equal number of traces below and above the nominal stimulus amplitude contribute to the average. Here, averaging across nine adjacent traces was found to be optimal.

One limitation of this study is that it is based on a single data pool. For the validation of the algorithm, one would ideally use a subset of data for training (optimizing parameters) and a different subset for independently evaluating the performance of the optimized algorithm [23]. The focus in our study was on understanding the effects of different parameters on FG-SNR formalism, defining useful parameter ranges, and obtaining indications for optimization. It would also be interesting to vary parameters simultaneously in future research to understand how parameters affect and influence each other. Future experiments could evaluate the expected clinical benefits of the FG-SNR algorithm, i.e., testing the minimum number of necessary above-threshold recordings of the algorithm and the potentially positive effects on measurement comfort (i.e., decreased loudness) and/or measurement duration. In addition, studying the performance of the FG-SNR approach in CI users with unusual ECAP morphologies will be of interest for clinicial application, as well as comparing this paradigm with other automated ECAP threshold determination methods.

Recently, it has been suggested to apply certain terms from error analysis to ECAP threshold determination, more specifically: a quantity, which is suitable to describe the error around the estimated threshold value [24]. The authors focused on two methods that are based on the amplitude growth function: (1) threshold determination by using linear extrapolation and (2) manual determination of the first visual ECAP response from all curves that were recorded. Confidence intervals around the threshold estimates were derived by extrapolating the 95% confidence interval around the linear fit (for the linear extrapolation method) or by using guidelines (for the “first visible” approach) [24]. We agree with the authors that ECAP amplitudes do have a measurement error and defining that error might help to clarify the mismatch between ECAP thresholds and behavioral thresholds. However, with the FG-SNR approach not being based on an amplitude growth function, we were unable to use a similar strategy. Nevertheless, we wanted to explore the value of a precision term to ECAP thresholds determined by the FG-SNR algorithm. To that end, we evaluated the precision of the sigmoidal function fitting-based threshold determination by applying the bootstrapping approach to determine the 95% confidence intervals [25]. The procedure was first performed separately for different CI users and for different stimulating electrodes, and then averaged across the individual CI users of the FLEX28 electrode array (i.e., the largest electrode population in this study). The analysis revealed that the 95% confidence intervals of the threshold estimates are maximally ± 1.24 nC wide (mean: 0.59; SD: 0.21), which makes the algorithm suitable also for discovering clinically relevant differences, e.g., in test-retest studies [26–30].

Therefore, even without the possibilities for further improvements that were discussed here, the concept of FG-SNR approach is suitable for ECAP classification and threshold determination. The algorithm is also hardware-independent and can be used with all CIs of different manufacturers. The only requirement is that the (custom made) research software can implement the FG stimulation paradigm [7]. Furthermore, we also deem the algorithm to be applicable in principle for predicting thresholds of other neural responses, such as cortical responses captured via electro- or magnetoencephalography (EEG and MEG, respectively) measurements, upon adjusting the parameters according to the known neurophysiological properties of the given target. Together, these aspects make the FG-SNR algorithm an intriguing tool for research on the neural responses elicited by CIs.

## Conclusion

A data processing pipeline was defined and successfully implemented in order to extend the signal-to-noise ratio (SNR) approach [11] for utilization with the FineGrain (FG) stimulation paradigm [7] for electrically evoked compound action potential (ECAP) threshold determination. This is called the FG-SNR approach. The outcome ECAP thresholds of this novel approach were evaluated versus the assessments of six evaluators. The ECAP thresholds estimated with the FG-SNR algorithm were found to be representative of the evaluators’ judgement, effectively demonstrating the use of this algorithm in clinical applications. The FG-SNR algorithm is not limited to any particular cochlear implant (CI) brand and can principally be applied for research purposes to any CI system by any manufacturer. Several parameters of the data processing pipeline were identified as promising points for further optimization of the FG-SNR formalism.

## Supporting information

S1 Appendix Details of the FG-SNR formalism. This document describes the data processing pipeline (DPP) in detail and the assigned parameters needed for realization of the FG-SNR approach.
(DOCX)

S1 Table Results of ECAP response classification by the evaluators and the FG-SNR algorithm and Pearson’s correlation coefficient r between evaluators and FG-SNR algorithm. Column A designates the parameter which was varied, and column B relates to the specific setting of this parameter. A parameter used as baseline is highlighted in green. Columns D–G show in how many cases the evaluator and the FG-SNR algorithm found an ECAP response (“true”). Column H shows Pearson’s correlation coefficient *r* for the correlation between the ECAP threshold estimates of the evaluators and the FG-SNR algorithm. Columns I, J and K show the average, median and standard deviation values of the *r* values across different evaluators, respectively. Empty cells indicate that the correlation could not be computed.
(XLSX)

S2 Table Detailed results of evaluators and FG-SNR algorithm. Sheets “FG-SNR ZAT”, “FG-SNR Denoising”, “FG-SNR Number of averages”, “FG-SNR Time window”, “FG-SNR Post-processing”, “FG-SNR SNR threshold” and “FG-SNR 2D filter” contain the ECAP threshold estimates obtained by applying the FG-SNR algorithm with different options of its parameters. In each of those sheets, column D describe the parameter that is modified and column E shows the option, while column G shows the ECAP threshold estimate. Empty values denote cases where no ECAP threshold was determined by the FG-SNR algorithm. Sheet “Evaluators” contains the ECAP presence classifications (column E) and ECAP threshold estimates given by the different evaluators.
(XLSX)

S3 Table Data underlying Fig 4. These Excel Sheets show underlying data shown in Fig 4A–4C.
(XLSX)

## References

[1] Cafarelli Dees D, Dillier N, Lai WK, Wallenberg E, Dijk B, Akdas F, et al. Normative findings of electrically evoked compound action potential measurements using the neural response telemetry of the Nucleus CI24M cochlear implant system. Audiol Neurootol. 2005; 10(2): 105–116. doi: 10.1159/000083366 15650302

[2] Lai WK, Dillier N. A Simple Two-Component Model of the Electrically Evoked Compound Action Potential in the Human Cochlea. Audiol Neurootol. 2000; 5(6): 333–345. doi: 10.1159/000013899 11025333

[3] Heyning P, Arauz SL, Atlas M, Baumgartner WD, Caversaccio M, Chester-Browne R, et al. Electrically evoked compound action potentials are different depending on the site of cochlear stimulation. Cochlear Implants Int. 2016 Nov; 17(6): 251–262. doi: 10.1080/14670100.2016.1240427 27900916

[4] Vaerenberg B, Smits C, De Ceulaer G, Zir E, Harman S, Jaspers N, et al. Cochlear implant programming: a global survey on the state of the art. ScientificWorldJournal. 2014; 2014(Article ID 501738): 12 pages. doi: 10.1155/2014/501738 24688394 PMC3932199

[5] He S, Teagle HFB, Buchman CA. The Electrically Evoked Compound Action Potential: From Laboratory to Clinic. Front Neurosci. 2017; 11: 339. doi: 10.3389/fnins.2017.00339 28690494 PMC5481377

[6] Botros A, Dijk B, Killian M. AutoNRT^™^: An automated system that measures ECAP thresholds with the Nucleus^®^ Freedom^™^ cochlear implant via machine intelligence. Artif Intell Med. 2007 May; 40(1): 15–28. doi: 10.1016/j.artmed.2006.06.003 16920343

[7] Gärtner L, Lenarz T, Büchner A. Fine-grain recordings of the electrically evoked compound action potential amplitude growth function in cochlear implant recipients. Biomed Eng Online. 2018 Oct; 17(1): 140. doi: 10.1186/s12938-018-0588-z 30340590 PMC6195717

[8] Strahl S, Dierker A, Spitzer P, Schwarz K. AutoART–A system for automatic determination of eCAP thresholds. In 21. Jahrestagung der Deutschen Gesellschaft für Audiologie; 2018.

[9] Litovsky RY, Goupell MJ, Kan A, Landsberger DM. Use of Research Interfaces for Psychophysical Studies With Cochlear-Implant Users. Trend Hear. 2017; 21: 1–15. doi: 10.1177/2331216517736464 29113579 PMC5764139

[10] Undurraga JA, Carlyon RP, Wouters J, Wieringen A. Evaluating the Noise in Electrically Evoked Compound Action Potential Measurements in Cochlear Implants. IEEE Trans Biomed Eng. 2012 Jul; 59(7): 1912–1923. doi: 10.1109/TBME.2012.2194292 22510942

[11] Hoth S, Spitzer P, Praetorius M. A new approach for the determination of ECAP thresholds. Cochlear Implants Int. 2018 Mar; 19(2): 104–114. doi: 10.1080/14670100.2017.1402472 29161976

[12] Marquardt DW. An algorithm for Least-Square Estimation of Nonlinear Parameters. J Soc Ind Appl Math. 1963; 11(2): 431–441.

[13] Laird NM, Ware JH. Random-Effects Models for Longitudinal Data. Biometrics. 1982; 38(4): 963–974. doi: 10.2307/2529876 7168798

[14] McCullagh P, Nelder JA. Generalized Linear Models. 2nd ed.: Chapman and Hall; 1989.

[15] Jacqmin-Gadda H, Sibillot S, Proust C, Molina JM, Thiébaut R. Robustness of the linear mixed model to misspecified error distribution. Comput Stat Data Anal. 2007; 51(10): 5142–5154. doi: 10.1016/j.csda.2006.05.021

[16] Shapiro SS, Wilk MB. An Analysis of Variance Test for Normality (Complete Samples). Biometrika. 1965; 52(3/4): 591–611. doi: 10.2307/2333709

[17] Holm S. A Simple Sequentially Rejective Multiple Test Procedure. Scand J Stat. 1979; 6(2): 65–70.

[18] Hosmer DW Jr., Lemeshow S, Sturdivant RX. Assessing the Fit of the Model. In Applied Logistic Regression. 3rd ed.: Wiley; 2013. p. 173–181.

[19] Brill S, Müller J, Hagen R, Möltner A, Brockmeier SJ, Stark T, et al. Site of cochlear stimulation and its effect on electrically evoked compound action potentials using the MED-EL standard electrode array. Biomed Eng Online. 2009 Dec; 8(1): 40. doi: 10.1186/1475-925X-8-40 20015362 PMC2803480

[20] Glassman EK, Hughes ML. Determining electrically evoked compound action potential thresholds: a comparison of computer versus human analysis methods. Ear Hear. 2013; 34(1): 96–109. doi: 10.1097/AUD.0b013e3182650abd 22885406 PMC3511653

[21] Gärtner L, Klötzer K, Lenarz T, Scheper V. Correlation of Electrically Evoked Compound Action Potential Amplitude Growth Function Slope and Anamnestic Parameters in Cochlear Implant Patients—Identification of Predictors for the Neuronal Health Status. Life. 2021; 11(3): 203. doi: 10.3390/life11030203 33807687 PMC7999542

[22] Gärtner L, Lenarz T, Joseph G, Büchner A. Clinical use of a system for the automated recording and analysis of electrically evoked compound action potentials (ECAPs) in cochlear implant patients. Acta Otolaryngol. 2010 Jun; 130(6): 724–732. doi: 10.3109/00016480903380539 19958247

[23] Park SH, Han K. Methodologic guide for evaluating clinical performance and effect of artificial intelligence technology for medical diagnosis and prediction. Radiology. 2018 Mar; 286(3): 800–809. doi: 10.1148/radiol.2017171920 29309734

[24] Biesheuvel JD, Briaire JJ, Frijns JHM. The Precision of eCAP Thresholds Derived From Amplitude Growth Functions. Ear Hear. 2018; 39(4): 701–711. doi: 10.1097/AUD.0000000000000527 29219858

[25] Wichmann FA, Hill NJ. The psychometric function: II. Bootstrap-based confidence intervals and sampling. Percept Psychophys. 2001; 63(8): 1314–1329. doi: 10.3758/bf03194545 11800459

[26] Björsne A, Magnusson L. When Can Stable AutoNRT Thresholds be Expected? A Clinical Implication When Fitting Young Children. J Am Acad Audiol. 2020 Jan; 31(1): 69–75. doi: 10.3766/jaaa.18077 31241451

[27] Christov F, Munder P, Berg L, Bagus H, Lang S, Arweiler-Harbeck D. ECAP analysis in cochlear implant patients as a function of patient’s age and electrode-design. Eur Ann Otorhinolaryngol Head Neck Dis. 2016 Jun; 133 Suppl 1: S1–S3. doi: 10.1016/j.anorl.2016.04.015 27262349

[28] Tanamati LF, Bevilacqua MC, Costa OA. Longitudinal study of the ecap measured in children with cochlear implants. Braz J Otorhinolaryngol. 2009; 75(1): 90–96. doi: 10.1016/s1808-8694(15)30837-5 19488566 PMC9442250

[29] Telmesani LM, Said NM. Electrically evoked compound action potential (ECAP) in cochlear implant children: Changes in auditory nerve response in first year of cochlear implant use. Int J Pediatr Otorhinolaryngol. 2016 Mar; 82: 28–33. doi: 10.1016/j.ijporl.2015.12.027 26857311

[30] Sainz M, Torre Á, Roldan C, Ruiz JM, Vargas JL. Analysis of programming maps and its application for balancing multichannel cochlear implants. Int J Audiol. 2003 Jan; 42(1): 43–51. doi: 10.3109/14992020309056084 12564515

