# Supplementary material for: Optimized SNR-based ECAP threshold determination is comparable to the judgement of human evaluators
Source: PLoS One. 2021 Nov 1;16(11):e0259347. doi: 10.1371/journal.pone.0259347 (PMC8559956; doi:10.1371/journal.pone.0259347)
Supplement: S1 Appendix — This document describes the data processing pipeline (DPP) in detail and the assigned parameters needed for realization of the FG-SNR approach. (DOCX) [file pone.0259347.s001.docx]

# Details of the FG-SNR formalism

Herein the authors provide a detailed description of the data processing pipeline (DPP) and the assigned parameters needed for realization of the FG-SNR approach, including the optimizing of its parameters. The description is organized according to the consecutive steps of the DPP.

## Consistency check

The input for the FG-SNR algorithm consists of a set of raw (unfiltered) single ECAP traces for each stimulus (separately for anodic- and cathodic-leading stimuli). Each curve is checked for consistency. All expected data must be available, and the curve must be within the specified linear signal processing range of the implant, i.e., without clipping or saturation of the built-in amplifier. Single curves that do not fulfill the consistency criteria are flagged and are treated specifically (e.g., they are omitted) by upcoming processing stages. This step requires no choice of parameters.

## Artifact reduction

The FG stimulation paradigm uses biphasic stimuli of alternating polarity for artifact reduction. Responses from anodic- and cathodic-leading pulses are averaged in pairs. No parameters are adjustable in this step.

## Zero amplitude template (ZAT) subtraction

The switch-on artifact of an amplifier operating at zero amplitude input is compensated by subtracting a zero amplitude template (ZAT) from every measured ECAP trace of the recording. The following ZAT options were investigated:

1. No ZAT subtraction
2. ZAT-A1
   Subtraction of the ZAT which is composed of the averaged curves from 0 to 1 nC.
3. ZAT-A3
   Subtraction of the ZAT which is composed of the averaged curves from 0 to 3 nC.
4. ZAT-A5 = baseline parameter
   Subtraction of the ZAT which is composed of the averaged curves from 0 to 5 nC.
5. ZAT-A7
   Subtraction of the ZAT which is composed of the averaged curves from 0 to 7 nC.
6. ZAT-F5
   The ZAT-A5 is approximated by a 2nd order polynomial function, which in turn is used as the ZAT.
7. ZAT-F0
   The ZAT without any averaging, i.e., at 0 nC, is approximated by a 2nd order polynomial function, which in turn is used as the ZAT.

## Noise reduction

- 1. Two-step approach
     1. Denoising (temporal filter)

According to the power spectrum of a noise-free mathematical model of a human ECAP signal [1], it is known that no spectral components above approximately 3 kHz have to be considered. Hence, filtering of high frequencies should not affect the ECAP response but would eliminate disturbing interferences. In this step, the type of filter and edge frequency are the parameters to be varied. The following filters were evaluated:

1. No filter
2. FIR-LP 3k = baseline parameter
   28^th^ order finite impulse response (FIR) lowpass filter with hamming window and 3 kHz cutoff frequency constituting a linear phase filter.
3. BW-LP 3k
   1st order Butterworth lowpass filter with 3 kHz cutoff frequency (-3 dB attenuation).
4. BW-BP 0.15/3k
   1st order Butterworth bandpass filter with a passband between 150 Hz and 3 kHz was selected (-3 dB attenuation).
   - 1. Moving average

We compared the effect of averaging different numbers of ECAP response curves over adjacent stimulus intensities as follows:

1. No averaging (*n* = 1)
2. Averaging 3, 5 (= baseline condition), 7, …, 33 adjacent curves, respectively.
   1. Two-dimensional filter (2D) approach

Instead of performing denoising and averaging separately, a two-dimensional filter could be used for noise reduction (e.g., as seen in image processing [2]) with the dimensions of time and stimulus amplitude. No parameters were adjusted in this step. When the ECAP traces are represented as “image” (2D matrix) with one axis being time and the other axis the stimulus amplitude, and the “color” (matrix elements) the response signal amplitude, a 2D filter can be used to reduce the noise (i.e., “blur” the image, [2]) instead of handling the axes separately.

## Time windows of “signal + noise” and “noise only” parts

Three different couples of time windows were applied:

1. separated = baseline condition
   The time window that contains “signal + noise” was chosen to be from *t*_1_ = 195 µs to *t*_2_ = 895 µs after stimulus onset. With a separation of 200 µs, the “noise only” part was chosen to be between *t*_3_ = 1095 µs and *t*_4_ = 1795 µs.
2. adjacent
   Similar to the original SNR approach [3], no temporal separation between the “signal + noise” and “noise only” parts was introduced. The theoretical time windows are *t*_1_ = 95 µs, *t*_2_ = 995 µs, *t*_3_ = 995 µs, *t*_4_ = 1895 µs, but effectively *t*_1_ and *t*_4_ were determined by the measurement delay that was applied. The “split” time point was chosen to be 995 µs after stimulation onset – this is exactly in the middle of the recording window (recording window duration is 1707 µs) for measurement delays of 145 µs. The two time windows cover the whole recording window, apart from 50 µs at the beginning and at the end of the recording window that were excluded in order to omit ringing artifacts. In the case of our study group, that meant *t*_1_ was always 195 µs (145 + 50), except for participant P09 where it was 175 µs (125 + 50); *t*_4_ is mostly 1802 µs (145 + 1707 – 50) except for participant P09, where it is 1782 µs (125 + 1707 – 50).
3. Gaussian
   Instead of using rectangular time-window functions, Gaussian tapered windows were chosen to separate the two regions smoothly. The center positions of the Gaussian windows corresponded to the center positions of the rectangular time windows (from the baseline condition) and the widths of the Gaussian windows (defined to be 2 sigma) were set to match the durations of the corresponding regions in the baseline condition. The Gaussians were applied within *t*_1_ = 195 µs (except P09 with 175 µs), *t*_2_ = 1802 µs (except P09 with 1782 µs), *t*_3_ = *t*_1_, *t*_4_ = *t*_2_.

## Post-processing “signal + noise” and “noise only” parts

To reduce any residual remaining stimulus artifact, which was not eliminated in previous steps, four different fitting approaches to the stimulation artifact were explored.

The original SNR approach used the exponential function [3]. However, since this fit is nonlinear, calculation time is high, the algorithm can fail to converge, or the fitting can result in extreme values for the fitted coefficients (unpublished observation by the authors).

1. No post-processing
   That leads to identical results since using an offset fit (polynomial 0th order) as the standard deviation does not depend on the mean.
2. poly1 = baseline condition.
   1st order polynomial (tilted line)
3. poly2
   2nd order polynomial (parabola).
4. exp
   Exponential function analogous to [3].

## SNR-based classification

In order to identify the signal within the “signal + noise” part, the quotient of the variances of the “signal + noise” and the “noise only” parts must exceed a certain threshold criterion, *q*_0_ (see Eq. 1 in the main document).

Different ratio cutoffs were tested:

1. *q*_0_ = 6.0 dB = baseline criterion
2. *q*_0_ = 1.5, 3.0, and 4.5 dB (less rigorous criteria)
3. *q*_0_ = 7.5, 9.0, and 10.5 dB (strict criteria)

## ECAP threshold determination

Ideally, all traces above a certain stimulus charge would be classified as “containing signal”, whereas no signal would be found below this limit. However, around the threshold, where the probability for the occurrence of a response equals 0.5, the decisions with respect to the presence of a response will often oscillate between 0 and 1. Therefore, it has to be defined how the SNR-based ECAP threshold should be derived from the single trace decisions. In the original SNR approach [3], a sigmoidal discrimination function was used to determine the threshold. No parameters were adjusted in this step.

# Bibliography

| 1. | Strahl SB, Ramekers D, Nagelkerke MMB, Schwarz KE, Spitzer P, Klis SFL, et al. Assessing the Firing Properties of the Electrically Stimulated Auditory Nerve Using a Convolution Model. Adv Exp Med Biol. 2016; 894: 143-153. |
| --- | --- |
| 2. | Burger W, Burge MJ. Digital image processing: an algorithmic introduction using Java. Second Edition ed.: Springer; 2016. |
| 3. | Hoth S, Spitzer P, Praetorius M. A new approach for the determination of ECAP thresholds. Cochlear Implants Int. 2018 Mar; 19: 104-114. |
